# Rediscovering life after being diagnosed with HIV: A qualitative analysis of lived experiences of youth living with HIV in rural Rwanda

**DOI:** 10.3389/frph.2022.993916

**Published:** 2022-11-04

**Authors:** Josée Uwamariya, Marcel Nshunguyabahizi, Jean Népomuscène Nshimyumuremyi, Gerardine Mukesharurema, Emmanuel Ndayishimiye, Innocent Kamali, Jean d’Amour Ndahimana, Bethany Hedt-Gauthier, Vincent K. Cubaka, Dale A. Barnhart

**Affiliations:** ^1^ Research Department, Partners in Health, Rwinkwavu, Rwanda; ^2^ Department of Global Health and Social Medicine, Harvard Medical School, Boston, United States

**Keywords:** HIV, youth, HIV status disclosure, orphans and vulnerable children, Rwanda

## Abstract

**Introduction:**

In sub-Saharan Africa, youth living with HIV, especially those who have lost one or both parents, face economic, socially and psychological challenges that hinder adherence to ART, ultimately leading to poor health outcomes. Partners In Health/Inshuti Mu Buzima implemented an Adolescent Support Group (ASG) to support HIV-positive youth aged 15–25 years. During the evaluation of the ASG program, we sought to better understand youths' lived experiences to improve our delivery of HIV care.

**Methods:**

We conducted qualitative in-depth, semi-structured individual interviews with youth enrolled in the ASG program. All interviews were conducted in-person or by telephone. Thematic analysis applying the framework approach with parallel inductive coding in Kinyarwanda and English was used.

**Results:**

We interviewed 35 youth who ranged in age from 16 to 29 years. The main themes related to the lived experiences of youth were (a) Experiences living with HIV, including disclosure, stigma, interactions with the health care system, and medication adherence; (b) external challenges, defined as challenges that were not related to the implementation of the ASG program; and (c) personal vision. Almost all youth reported acquiring HIV from their mothers and disclosure of HIV status occurred around the age of 10. Disclosure was often unintentional and followed by internalized and enacted stigma. Many reported poor past medication adherence which improved following enhanced counselling. External challenges were overwhelmingly economic in nature, and orphanhood was a root cause of other challenges such as difficulty accessing education, lack of transport to health facility, and lack of insurance fees. Despite these challenges, youth have an optimistic view of the future with dreams of health, economic attainment, marriage, and children.

**Conclusion:**

Healthcare providers should empower caregivers to support HIV disclosure. Supporting youth as they face many economic challenges could help address socio-economic barriers to good health and promote holistic well-being.

## Introduction

In 2019, approximately 3,4 million youth between the ages of 15 and 24 years were living with HIV globally (1)*.* These youth are less likely than older adults to get tested for HIV, to adhere to treatment plan and to be retained in care (2). Worldwide, HIV mortality reduced by 39% in all age categories from 2010 to 2019, however the prevalence rise by almost 25% among youth (3). Youth living with HIV are susceptible to social stigma, anxiety regarding the future, insufficient social support, and have a fear of HIV status disclosure, leading to profound experiences of internalized stigma (4). Ultimately, these dynamics often lead to poor health, including poor HIV-related outcomes, such as poor adherence to treatment plan, high viral loads, and high loss to follow-up, as well as poor mental health outcomes, including depression, lack of self-acceptance, and low self-esteem (5).

However, youth's clinical outcomes can vary based on contextual factors. In Africa, where HIV is the fifth leading cause of death among male youths and second among female youth (1), recognized contextual factors hindering good health among youth with HIV include living in extended families where children have less privacy to take medication; long distances to health facilities and lack of resources for transportation; limited availability of healthcare providers trained in delivering youth-friendly services, and ineffective disclosure leading to poor psychological outcomes (6, 7). Rwanda has made great strides in promoting widespread access to ART and improving the quality of HIV services to ensure that patients have improved adherence and retention (8). However, adherence is still a documented challenge among adolescents aged 10 to 17, partly due to psychological symptoms such as depression (9). Many of these challenges likely persist among older youth.

To better support youth living with HIV, Partners In Health/Inshuti Mu Buzima (PIH/IMB) implemented Adolescent Support Groups (ASGs), a complex intervention that included both peer support groups and group-based economic incentives, in health facilities in rural Rwanda. Despite its name, this program did not only target adolescents and was instead designed to support youth 15–25. In the mixed-methods evaluation of this program, the clinical benefits were not statistically significant, although the youth members identified many non-clinical benefits and suggested many key areas for improvement (3, 10). However, during interviews with youth members, we also identified several key themes that were not specific to youths' experiences in the ASG program but provided more general insights into their experiences as young people living with HIV in rural Rwanda. This paper explores the ASG members' lived experiences, with a focus on their experiences living with HIV, challenges they face, and their personal vision. By better understanding these experiences, we hope to identify ways to improve delivery of HIV care for children and youth.

## Methods

### Study design

This is an explorative qualitative study using in-depth, semi-structured individual interviews with youth enrolled in the ASG program.

### Setting

In Rwanda, 84% of young people between the ages of 15–24 are literate (11). HIV prevalence remains stable around 3% in the general population and 1% among youth (12). In Rwanda, 85,6% of the population is insured by community based health insurance (CBHI), also known as mutuelle de santé (13). CBHI members pay a contribution of 3$ per year, however poor families' contribution is covered by the government and employees contribute 0.5% of their monthly net salary to the CBHI scheme (14). The community based health insurance (CBHI) policy and the provision of HIV treatment free of charge to all to positive people had increased the accessibility to HIV testing, diagnosis and treatment (15).

The ASG program was implemented in 34 rural health facilities in Burera, Kayonza and Kirehe districts, Rwanda. These health centers are supported by PIH/IMB, an international non-governmental organization that supports health system strengthening through collaborations that include constructing and staffing of hospitals and increasing access to HIV and TB treatment since 2005. PIH/IMB supports the HIV program of the three districts through mentorship of HIV nurses at primary health facilities and provision of transportation fees to help some HIV patients keep their appointments. In Rwanda, HIV care and treatment services are provided in 96% of all health facilities (16). Since 2016, ART has been prescribed to every HIV positive patients regardless of their CD4 count and pharmacy visits are currently scheduled monthly for new patients and every six month for patients >15 years who have been on ART for at least 18 months, demonstrated good adherence, and achieved viral load suppression (17).

### Description of the adolescent support program

The ASG program was designed and implemented by PIH/IMB in collaboration with health care providers at participating primary-level health facilities. The program was first launched in Kayonza in July 2017 and was scaled up to Burera and Kirehe in July 2018. Healthcare providers in charge of the HIV program were requested to select 10 to 15 economically vulnerable youth aged 15–24 years old to participate in the ASG program, giving a particular attention to orphans and school dropouts. The ASG program combines peer support and economic empowerment; the peer support aspect of the program involves organized monthly nurse-facilitated meetings where all group members convened and discussed medication adherence and social economic challenges. The economic empowerment aspect of the program involves providing incentives to the group based on the achievement of predefined three main indicators: (1) quarterly pharmacy visit attendance, (2) biannual achieving group savings targets and (3) annual viral suppression.

### Participant selection

Interviewed participants were youth living with HIV who had participated in an ASG. Youth were purposively sampled from over-performing ASGs, under-performing ASGs, or from among ASG dropouts, where group performance was evaluated retrospectively using data from an electronic medical record (EMR) system (10).

### Conducting interviews

Between June and early July 2021, interviews were conducted in-person and scheduled to coincide with regular ASG meetings and to avoid interrupting youth's usual daily activities. During the meeting, trained data collectors explained the purpose of the study and asked group members aged 15 years or older to voluntarily participate in one-on-one interviews anticipated to last between 45 min to 60 min. In each group, a maximum of three members were selected to participate in the interviews. If more than three members volunteered, we randomly selected three members by drawing slips of paper out of a container. Selected participants provided written consent and were immediately interviewed in a private room at the clinic. Data collectors used an interview guide that was designed based on a review of the literature and in collaboration with the PIH/IMB staff who implemented the ASG project.

After July, cross-district travel in Rwanda was restricted due to a surge of COVID-19, so we shifted to conducting telephone-based interviews. During telephone-based data collection, nurses' in charge of HIV at each health center provided our data collection team with phone numbers of ASG members or ASG dropouts. We generated a random calling order for each ASG group as well as for ASG dropouts and data collectors contacted the top three members by phone to explain the study and invite them to participate. If interested, we scheduled one-on-one phone interviews and the interviewee provided oral consent prior to the interview. All interviews were conducted in Kinyarwanda and audio-recorded.

### Data analysis

All interviews were transcribed and translated into English. A thematic analysis applying the framework approach was carried out (18). The research team, which included three native Rwandans who had conducted the interviews and one native English speaker (an American epidemiologist living in Rwanda), conducted all coding. No member of the research team had been involved in the implementation of the ASG or the youths' clinical care. We constructed a codebook using inductive parallel coding where each interview was independently coded by two coders, one using the Kinyarwanda transcript and the other using the English translations (19). Identified codes were discussed and harmonized during two full-team meetings, one occurring after the end of in-person data collection and the second occurring after the end of all data collection. After a codebook was developed, two team members conducted deductive coding on the English translations and made final revisions to the codebook as needed. Key demographic data was extracted in youth interviews to Excel to report medians, ranges or percent. The analysis process was facilitated by MAXQDA 2020 Software (20).

### Ethics

This study received approval from Inshuti Mu Buzima Research Committee (IMBRC), Rwanda National Ethics Committee (NO. 150/RNEC/2020) and Harvard University approvals (IRB20-0565). Informed consent were obtained for all adolescents who participated in the study. The ethic committees waived the requirement for caregiver consent for adolescents below 18 years old to avoid involuntary disclosure of adolescents' HIV status.

## Results

In total, we interviewed 35 participants. Seventeen interviews were conducted in-person and 18 over the telephone. The median age of respondents was 20 years (range: 16 to 29) and 20 (57%) were male. The main themes identified during the inductive coding that was linked to the lived experience of youth were (a) Experience living with HIV, (b) External challenges and (c) Personal vision.

### Experiences living with HIV

Subthemes explored within the main theme of experiences living with HIV included disclosure of HIV status, stigma, interaction with health systems, and medication adherence. Illustrative quotes are provided for each of these subthemes in Table 1. Although almost all youths reported acquiring HIV from their mothers, the median age at disclosure was 10 years. Some participants reported that their HIV status was disclosed to them by caregivers, but often only after youths persistently asked why they must take medications. Other youths had simply observed their parents' taking medication and gradually understood. In these cases, youth often felt lied to and resented that their parents had not told them the truth (Quote 1.1.1; 1.1.2). Many others reported that they learned their HIV status from health care workers during group meetings at the health center. These meetings were designed to provide counselling about medication adherence without providing specific support for status disclosure (Quote 1.1.3). A few participants had learned of their HIV status through voluntary testing either during community HIV screening or at the health center following diseases recurrence (Quote 1.1.4; 1.1.5).

**Table 1:** Sub-themes and illustrative quotes from the main theme, “Experiences living with HIV”.

Experience living with HIV
1. HIV disclosure	1.1.1. “When I was a toddler, I found myself taking medications. (When) I asked my mum why I always take medication, she provided some explanations. Later on I understood what she was trying to explain to me and I accepted myself. At the beginning I hated myself, I hated my mum because she transmitted the virus to me. But I finally understood that she didn’t want that it happens to me”
	1.1.2. “I didn’t learn that from my mum. I always took medicines and saw my mum and daddy taking medicines everyday as well. I asked myself what was the medication for because it wasn’t for my leg disease. After careful observation, I found out that it was for HIV”
	1.1.3. “We were in the counseling meeting here at the health center. I realized that the main topic we were discussing about is HIV. I started questioning my status. Then I asked the facilitator if all participants in the meeting have HIV. She replied, “Yes.” That's how I found out”
	1.1.4. “There was a community HIV screening, where whoever interested would get a voluntary test. I went alone without my mother,and tested positive”.
	1.1.5. “I was always sick. I lost weight constantly and felt too weak. I went to the health center, they told me that it is not malaria. The doctor tested me and advised me to start medications (ART)”.
2. Stigma	***Enacted stigma***
	1.2.1. “People were isolating me because they saw me always going with my mum to (the health center). They insulted me, but I didn’t know exactly what was going on”.
	1.2.2. “There is a time when a neighbor woman saw me discussing with a young man. She told that man about it (HIV). The man came back and asked me and didn’t know how to reply, I just kept quiet. I stopped ARV for two months and tried to commit suicide”
	***Internalized -stigma***
	1.2.3. “First of all, before I joined the group I had no self-acceptance, I felt like life is over, I had no hope for the future. Most of the time I used to stay in my room, and even when I went out, I put on my headphones so that no one will talk to me”
	1.2.4. “I don’t have challenge (taking medication) except when I am in a place where there are a lot of people, you know it is a personal secret that need to be protected. So when I am in a place with a lot of people, I have to hide them and take it while no one sees me”
	1.2.5. “At the beginning, I felt like the world was overflowing with me because I thought I was the only one to have this problem”
3. Interaction with health system	1.3.1. “The health care service is well delivered; they treat us well. And if there is an event involving youth, we are the first ones to be invited to participate in the implementation of that event. Inviting us into different events, it is evidence that they value us and consider as not only as patients seeking healthcare but as valuable stakeholders”
	1.3.1. “He (the nurse) discusses with us in a friendly manner, he advises us, he comforts us in a way that motivates us to open up to him”.
	1.3.2. “The service at the health center is so good and we receive it on time. We are happy for our new nurse, we can call him any time, and he calls us regularly to learn how we are doing and the challenges we are facing. We feel comfortable to call him if we have problems”
	1.3.3. “The service we receive at the health center is so amazing, they give us regular appointments and we don’t experience shortages of medication”
4. Medication adherence	***Poor adherence***
	1.4.1. “In the past, I used to skip doses and miss some appointment because I was angry and I was afraid that people would see me (at the ARV service) and raise suspicion (about my HIV status)”
	1.4.2. “There were times when I used to forget (to take ARV), or not take them on time. But currently, I take them without any problem”.
	1.4.3. “Forgetting to take medications happened to me when I was a child because I didn’t fully understand. But now that they have taught me the benefits of taking medication as recommended, it no longer happens”.
	1.4.4. “ (Skipping medication doses) really happens to me. When I have an empty stomach, taking the pill leads to side effects”.
	***Good adherence***
	1.4.5. “Now it is not difficult to take (ART). It was difficult when they gave us pills that would last for one month. Currently the give us quarterly medication and I have no problem with that”.
	1.4.6. “It has never happened. Since my parents are always there for me and they have HIV too, we remind each other. I have never missed even a single day”.

HIV disclosure was often followed by enacted stigma within families and communities. Youths reported being isolated and mistreated once the community became aware of their HIV status (Quote 1.2.1). This enacted stigma resulted in internalized stigma, where youths hated themselves, leading to poor mental health outcomes, including hopelessness, self-reported depression and suicidal ideation (Quote 1.2.2). Internalized stigma also led to a lack of self-acceptance (Quote 1.2.3). Although many youths reported that they have grown to accept their HIV status, they still said that HIV status is a personal secret that needs to be protected and often did not feel comfortable taking medication in front of others (Quote 1.2.4). Similarly, in our interviews they avoided describing themselves as people living with HIV, instead saying things like they “live with this problem” (Quote 1.2.5).

Though ASG members have experienced enacted and internalized stigma, their interactions with the health system were generally positive. Youths mentioned that the health centers consider them to be valuable stakeholders and involve them in different youth-related projects (Quote 1.3.1). Many youths emphasized good relationships with their nurses and felt comfortable discussing their treatment plan as well as medication related challenges (Quote 1.3.2; 1.3.3). Youths appreciated the timely service that they receive and recognized that they do not experience shortages of medication (Quote 1.3.4). The good relationship with nurses and timely service motivated youth to develop healthier behaviors including appointment keeping and medication adherence.

Many youths reported that their medication adherence had been bad in the past and that they used to skip medication doses and miss medical appointment as a result of lack of self-acceptance or fear of people seeing them at the HIV clinic (Quote 1.4.1). Some youth also simply forgot to take medication. Generally, education on the importance of medication adherence had improved their adherence and currently they take their medications as prescribed (Quote 1.4.2, 1.4.3). Although the current adherence situation had generally improved, youth reported that it was still very hard to take ARV medication when they have an empty stomach because it induces side effects (Quote 1.4.4). Other facilitators of medication adherence included multi-month prescriptions (Quote 1.4.5) and parental support (Quote 1.4.6).

### External challenges

External challenges, defined as challenges that were not related to the implementation of the ASG program, were overwhelmingly economic in nature (Table 2). They included orphanhood, difficulty accessing education, unstable housing, family conflict, supporting impoverished family members, food insecurity, lack of transport to health facilities, and lack of insurance fees. The majority of the interviewed youth had lost one or both parents. Orphanhood was a major root cause of socio-economic challenges, causing some youth to live alone and provide for themselves financially (Quote 2.1.1; 2.1.2).

**Table 2:** Sub-themes and illustrative quotes from the main theme, “external challenges”.

External challenges
1. Orphanhood	2.1.1. “I live alone at home; my parents died”
	2.1.2. “The only thing that make us different (from each other) is that some are orphans. Those who don’t have parents experience some hardship as they have to take care of themselves”.
2. Difficulty accessing education	2.2.1. “I went to school and completed only the primary level. I dropped out early because my parents passed away”.
	2.2.2. “I spend most of my time studying. Even when I reach at home I revise. I wish I could get someone to help me scholastic materials that I need”.
	2.2.3. “The thing we need most is education. Because education is a sustainable investment… For members who dropped out of school early, they can help them get enrolled in vocational training. Those enrolled in school, sometimes their parents are struggling to get school fees, health insurance, school books, uniforms, hence they miss school for one week due to insufficient financial abilities”.
3. Unstable housing	2.3.1. “Though you see them here, most of them are homeless, only 10% have a place to stay, Others are like nomads, they keep moving from one place to another”.
4. Family conflict	2.4.1. “When I arrived home my stepmother starts telling me that my parents spread the virus to me in front of my youngest stepsiblings., consequently I don’t get along with them. I get home and find no food, or someone insults me, I immediately go to sleep and don’t take the medicine”.
5. Food Insecurity	2.5.1. “I can’t miss financial problems because I am poor. Sometimes I even miss a meal”.
6. Health insurance	2.6.1. “The problem I had, I didn’t have health insurance, but I didn’t tell my group because I knew that they were not going to help”
	2.6.2. “Recently I was ill, I have hemophilia disease which causes severe bleeding. Unfortunately, I didn’t have health insurance because my family was not able to pay for it. I was worried because if I happened to get a wound, I couldn’t get healthcare”.
	2.6.3. “Sometimes you get sick, for example I have recurrent stomach disease, I wish I would get support so that I may get proper treatment. I don’t have health insurance, however my adoptive dad told me that he will pay”
7. Transportation fees	2.7.1. “Recently I was referred to Butaro hospital but I didn’t get the transportation fees”
8. Supporting impoverished family members	2.8.2. “Sometimes youth came from poor families and used the money earned to fix family problems”.

Orphanhood was also associated with difficulty accessing education. Only few interviewed youths were still enrolled in school, with the majority having dropped out of primary school, and few others having dropped out of secondary school. Youth reported dropping out of school following the death of their parents because it was hard to get school fees or access other scholastic materials such as uniforms and books (Quote 2.2.1). Even youth who were still enrolled in school reported challenges accessing school fees and scholastic materials (Quote 2.2.2). Many youths recognized access to education as a sustainable investment and explicitly requested support to continue formal education or enroll in vocational training (Quote 2.2.3).

Many youths reported being homeless or living in unstable housing due to an inability to pay rent (Quote 2.3.1). Some youths with stable housing reported being in direct conflict with their caretakers, which consequently contributed to poor adherence to medication (Quote 2.4.1). Food insecurity was an additional challenge to youth, and this insecurity sometimes impacted their adherence (Quote 2.5.1). Youth coming from such extremely poor families reported having difficulty accessing health insurance and transportation fees, which affected their ability to seek care for both HIV and for other health issues (Quote 2.6.1; 2.6.2; 2.6.3; 2.7.1) Although youth often reported to experiencing economic hardships, they were also sometimes responsible for supporting impoverished families themselves (Quote 2.8.1).

### Personal vision

Youths' personal vision for the future was optimistic and positive (Table 3). Their long-term vision demonstrated that they no longer perceived HIV as a death sentence but instead as a chronic, manageable conditions that was compatible with a fulfilling life and dreams and goals. Many youths wish to develop economically, have stable jobs, and improve their health, with a particular focus on attaining an undetectable viral load (Quote 3.1; 3.2). Many youths want to invest in their education and become experts in their chosen careers (Quote 3.3) while others want to create their own business and generate income (Quote 3.4). Youth wanted to be financially independent not just to improve their own lives but also to impact lives of people in their community through providing support in career development (Quote 3.5). Besides their professional and career goals, many youths reported that they want to get married and build families (Quote 3.6).

**Table 3:** Illustrative quotes from the main theme, “personal vision”.

Personal Vision
Personal Vision	3.1 “I wish I will be more developed than today. Second, I wish that my viral load will be undetectable, and third I wish I will have something to do (a job)”.
	3.2 “I feel like having a good life with no problems. If I respect the doctor's appointments and take medications as recommended, these will make my life healthier in 5 years”.
	3.3 “I want to be a medical doctor. I wish I could get people who can pay school fees for me in the medical department”.
	3.4 “I could get a new laptop, I can start working for myself and save. I would use my first saving to buy pigs and build a house for them. But before I buy pigs, I would raise chickens”.
	3.5 “I want to be someone important, financially independent. I want to impact lives of people in my community by helping them find something to do”.
	3.6 “In the next 5 years I feel like getting married perhaps, and then give my wife what she wants”.

## Discussion

Our qualitative interviews among youth living with HIV in rural Rwanda revealed complex interactions between early negative experiences with HIV, poverty, and poor medication adherence. At the same time, our findings suggest that support and education from health care professionals can help youth understand the importance of medication adherence and to reframe HIV as a manageable chronic condition.

Many of our participants reported delayed disclosure of HIV status and expressed that disclosure did not occur in a private, age-appropriate manner. Appropriate disclosure of HIV has positive clinical and psychological benefits that promote the quality of life including improved adherence, viral load suppression, self-acceptance, and self-esteem (21). Currently, the WHO recommends that school age children and younger children should be informed of their HIV status incrementally to accommodate their psychological maturity while preparing them for full disclosure (22). The current Rwanda guideline on disclosure recommends that school age children from 7 years old should be notified of their HIV status in presence of their caretakers after a carefully cognitive and psychological assessment (17). However, HIV-positive caregivers are frequently reluctant to disclose their own HIV status to their children due to fear of negative consequences as well as due to worries that the child will be unable to understand and accept their diagnosis or will disclose the family's HIV status to others (23). Interventions aiming at empowering caregivers to effectively disclose HIV to perinatally infected youth should take into consideration beliefs and cultural factors in order to develop an intervention tailored specifically to that population (23).

Although most of the youth reported good adherence at the time of our interviews, they also described prior episodes of poor adherence, usually as a result of lack of self-acceptance or fear of discrimination. A similar patterns of HIV disclosure being followed by depression, isolation, and medication cessation have been reported elsewhere in East Africa (5). Periods of non-adherence to ART can lead to opportunistic infections, development of ART-resistant virus, and to progression to AIDS (24). Even if youth begin to adhere ART appropriately at a later date, these earlier periods of non-adherence could led to drug resistance and potentially explain the very high prevalence of drug resistance among Rwandan youth (25) as well as the low prevalence of viral load suppression among ASG members (26). Currently, the WHO recommends that people with high viral load count should receive 3–6 months enhanced adherence counseling (EAC) sessions before diagnosing virological treatment failure (27). However, for perinatally infected youth who may have historically had poor adherence but currently report appropriate treatment adherence, more proactive monitoring for ART resistance may be warranted.

Our study identified that many interviewed youths had lost one or both parents. As has been reported elsewhere, perinatally infected youth are especially likely to live in extreme poverty due to orphanhood of one or both parents (5), which can prevent them from accessing medical treatment for HIV though free of charge (28), cause them to drop out of school (29), and caust them to struggle to afford healthy food and stable housing (30). In 2010, Rwanda endorsed a policy to shut down all orphanages. Since then, Rwandan orphans were placed with their extended families or in foster families. In general, living with extended families likely provides an improved safety net for orphans and vulnerable children. However, for orphans living with HIV in these circumstances may also force the youth to face challenges surrounding disclosure, stigmatization, and inability to take medication due to poor perception of HIV among their extended families (31). Health care providers should be aware that, although extended family placements could make it less obvious which Rwandan children living with HIV are orphans, these orphaned children still constitute a particularly vulnerable population in need of economic and emotional support.

Despite these challenges, our study revealed that the youth perceived that the HIV services provided by the health center were high-quality, and reported that good relationship with their healthcare providers helped them to develop ownership over their treatment journey, ultimately resulting in improved adherence to treatment and retention in care. Previously evidence has similarly shown that patient satisfaction among patients living with HIV lead to improved health outcomes, including adherence to ART and retention to care (32) and that good relationships between the healthcare providers and HIV-positive patients yields improved patients' quality of life, self-esteem and adherence (33).

This study does have several limitations. Our primary limitation is that all interviews were conducted among ASG participants, who may not be representative of all HIV-positive youth in rural Rwanda. In particular, these youths may have more positive views of the health care system, better medication adherence, or improved future orientation compared to youth who did not received a comparable intervention. Similarly, interviewed youth were 15 years of age or older, and their childhood experiences may not reflect the current experience of HIV-positive children, especially as targeted training around disclosure of HIV status to children had been implemented in Rwanda in 2008 when our study participants would have been aged between 2 and 16 years. Additionally, we transitioned to telephone-based interviews due to a surge during the COVID 19 pandemic, so the poorest ASG members who lacked access to mobile phones may have been excluded from some interviews. Finally, there was a possibility of social desirability bias where respondents may have underreported socially undesirable behaviors and over reported desirable attributes because the interviewing team were from the organization that supports HIV care in the respondents' health centers and the implementation of the ASG project.

## Conclusions

Despite these limitations, our results highlight several challenges experienced by youths living with HIV and suggest a number of avenues to better support these youths. First, training healthcare providers to empower caregivers to appropriate disclosure HIV status to perinatally infected youth is a critical step for ensuring that children with HIV are prepared for success in their adolescence and beyond. Second, framing HIV as a manageable chronic condition and educating young people on the benefits of medication from an early age may help motivate youth to adhere, leading to better long-term health outcomes. Third, clinicians should recognize that perinatally infected youth could have developed antiviral resistance from prior periods of non-adherence, even if their current adherence is good, and should provide appropriate, compassionate counselling. Fourth, intentionally tracking adolescents' orphanhood status as part of their medical records and providing emotional and economic support is warranted, even when the child has been placed with an extended family. Providing these orphaned youths with community based-accompaniment, which has been previously demonstrated to improve health outcomes and mental health among adult HIV patients in Rwanda (34), may be one concrete strategy to ensure that the youth is receiving appropriate support from an adult. In order to address these issues in a systematic and sustainable manner, the government may also consider appointing an official to oversee outcomes among youth with HIV and implement improved training for health care providers providing HIV care that reinforces appropriate disclosure strategies, provides health education messaging for children and youth with HIV, and investigates a revision of drug resistance testing policies for perinatally-infected youth.

## Data Availability

The raw data supporting the conclusions of this article will be made available by the authors, without undue reservation.
